# Ferromagnetic resonance induced large microwave magnetodielectric effect in cerium doped Y_3_Fe_5_O_12_ ferrites

**DOI:** 10.1038/srep28206

**Published:** 2016-06-20

**Authors:** Fu Chen, Xian Wang, Yan Nie, Qifan Li, Jun Ouyang, Zekun Feng, Yajie Chen, Vincent G. Harris

**Affiliations:** 1 School of Optical and Electric Information, Huazhong University of Science and Technology, Wuhan, 430074, China; 2 Center for Microwave Magnetic Materials and Integrated Circuits, and Department of Electrical and Computer Engineering, Northeastern University, Boston, MA 02115, USA

## Abstract

In recent years, multifunctional materials contained simultaneous ferroelectric and ferromagnetic ordering have been realized. Here, a real time room temperature adaptive materials system, which demonstrates an RF magnetodielectric (MD) response, i.e., *Ce*_*x*_*Y*_*3−x*_*Fe*_*5*_*O*_*12*_ (*x* = *0, 0.05, 0.1, 0.15, 0.2*), is reported. The magnetic and dielectric properties of Ce-doped YIG microwave ferrites processed by a traditional ceramic route have been measured over a frequency range of *4–8* *GHz* (C-band). The substitution of Ce not only enhances the microwave electromagnetic properties of the YIG, but also modulates the magnetodielectric response. The maximum magnetodielectric response in Ce-doped YIG sample ranges in magnitude from approximately +*5%* to −*5%* under an applied field of *1.78 kOe.* This effect was attributed to electron fluctuations on the Fe cation sites. Furthermore, the magnitude of the MD response was shown to be enhanced by the cerium content. It is believed that research of the magnetodielectric effect in YIG ferrites is of great importance to the development of next generation multifunctional adaptive microwave materials, devices and integrated circuits.

With the rapid advances in modern communication technologies, it is now considered necessary to develop advanced materials that enable the design of novel multifunctional and real time adaptive devices and systems. Due to the rare coexistence of ferromagnetism and ferroelectricity in a common materials system, multiferroic (MF) materials with significant magnetoelectric (ME) or magnetodielectric (MD) effects have recently attracted lots of attentions. As early as the 1950’s, the phenomena of ME/MD effects in single-phase materials have been recorded^1^. Various single-phase materials have since been identified, such as *Ti*_*2*_*O*_*3*_^2^, *BiFeO*_*3*_^3^, *BiMnO*_*3*_^4^, *LuFe*_*2*_*O*_*4*_^5^, and garnet films^6^, among others. However, the level of research activity dedicated to these material systems declined due to the rare existence, and the relatively low ME/MD coupling in single phase materials. Owing to the large conversion between magnetic and electrical energy at room temperature, it is the multi-phase MF materials that have garnered more attentions and are believed to possess greater potential for multifunctional microwave device applications^7^,^8^. It has been confirmed that the MF composites combining piezoelectric (*BaTiO*_*3*_, PZT, PVDF, PMN-PT etc.) and magnetostrictive (*CoFe*_*2*_*O*_*4*_*, NiFe*_*2*_*O*_*4*_*, CuFe*_*2*_*O*_*4*_, Terfenol-D etc.) phases can achieve large ME coupling at room temperature^9^,^10^,^11^,^12^. Multi-phase MF materials are therefore considered to be the candidates for the next generation of multifunctional devices such as sensors, transducers, actuators filters, high frequency microinductors, and tunable microwave devices^13^,^14^,^15^,^16^,^17^,^18^. Although the multi-phase MF materials have made great progress, the studies of single-phase MF material remains meaningful since they possess intrinsic ME/MD coupling mechanisms that remain poorly understood today. In other words, the further understanding of the ME/MD effect in single-phase materials may prove effective ways in MF device development.

We propose that microwave materials and devices will play a leading role in positively impacting global markets in the microwave communications sector. Of particular interest are electrically-tunable microwave devices as real time adaptive magnetoelectric systems having low microwave loss and strong ME/MD coupling effects^19^,^20^,^21^. Yttrium iron garnet *Y*_*3*_*Fe*_*5*_*O*_*12*_ (YIG) and its variants are the most popular candidates due to their low microwave loss, high resistivity, and structural & chemical stability. Moreover, ME/MD coupling has been observed in YIG ferrites^2^,^6^,^22^ and its various dopant systems, such as *La*_*x*_*Y*_*3−x*_*Fe*_*5*_*O*_*12*_, *Yb*_*x*_*Y*_*3−x*_*Fe*_*5*_*O*_*12*_ and *Y*_*3*_*Fe*_*5−x*_*Ti*_*x*_*O*_*12*_ etc.^23^,^24^,^25^. The existence of the ME/MD effect in the YIG-based ferrites may prove to be a suitable materials platform for the investigation of the intrinsic mechanism of ME/MD effect.

In this work, we present C-band dynamic MD behavior induced by FMR in a well-known magneto-optical material: *Ce*_*x*_*Y*_*3−x*_*Fe*_*5*_*O*_*12*_ (Ce-doped YIG) ferrite. The effect of Ce ions upon the MD response in this system has also been discussed in order to investigate the origin and underlying mechanisms responsible for the MD effect.

## Results

**Figure 1:**
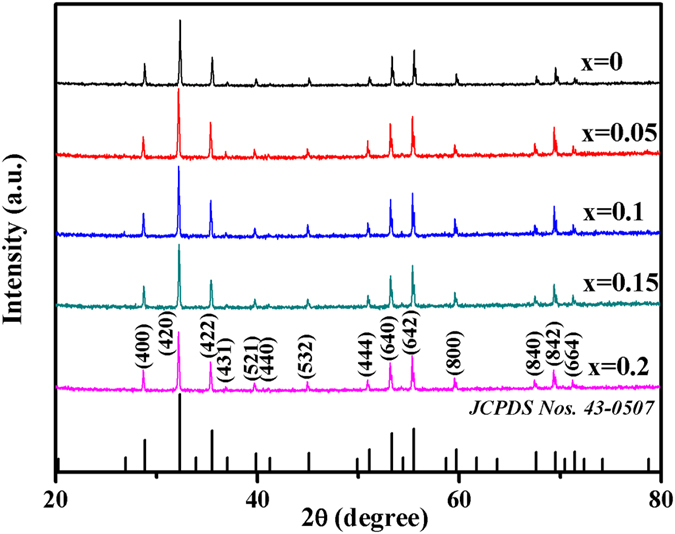
The XRD patterns of the Ce-doped YIG ferrites.

X-ray diffraction (XRD) patterns of *Ce*_*x*_*Y*_*3−x*_*Fe*_*5*_*O*_*12*_ *(x* = *0, 0.05, 0.1, 0.15, 0.2)* ferrites are shown in Fig. 1. All diffraction peaks of the Ce-doped YIG samples have been indexed to the standard powder diffraction pattern of pure YIG (*JCPDS Nos 43-0507*). It has been confirmed that all ferrites samples exhibit a pure garnet phase. Based upon the XRD patterns, the calculated lattice constants increase from *12.38* *Å* to *12.42* *Å* with the substitution of cerium ions. In comparison to the radius of Y^3+^ (0.9 Å), the lattice expansion is attributed to the larger radius of Ce^3+^ (1.02 Å).

**Figure 2:**
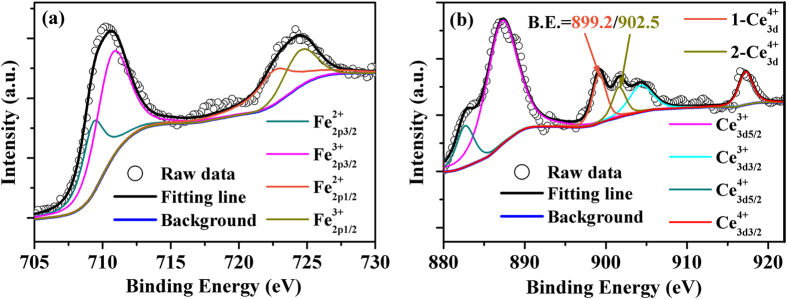
The XPS spectra of (**a**) Fe and (**b**) Ce in *Ce*_*x*_*Y*_*3−x*_*Fe*_*5*_*O*_*12*_ *(x* = *0.2)* ferrite.

It is inferred that the Ce-doped YIG ferrites likely contain Fe^2+^ ions due to the low oxygen condition during high temperature sintering. This assumption has been verified by the XPS spectra of *Ce*_*x*_*Y*_*3−x*_*Fe*_*5*_*O*_*12*_ *(x* = *0.2)* ferrite as shown in Fig. 2. The 2p_3/2_ and 2p_1/2_ peaks of the Fe ion can be observed at 710.6 eV and 724.3 eV in Fig. 2(a) respectively. These features are located between the corresponding values obtained for pure *Fe*_*2*_*O*_*3*_ and *Fe*_*2*_*SiO*_*4*_ which are considered to contain only Fe^3+^ and Fe^2+^ ions, respectively^26^. Similar phenomena in the binding energy shift for 2p_3/2_ and 2p_1/2_ peaks can also be found in pure Fe_3_O_4_ and other oxides that contain both Fe^2+^ and Fe^3+^ ions^23^,^27^. In addition, the 2p_3/2_ peak of Fe ions in Ce-doped YIG ferrite can be divided into two peaks located at 710.8 eV for 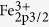 and 709.5 eV for 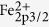 as determined by fitting of the spectra using Lorentzian-Gaussian method. Figure 2(b) shows a typical 3d spectrum of Ce^3+^ and Ce^4+^ ionic mixture^28^. Characteristic peaks of the 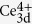 ion can be observed at 899.2 and 902.5 eV, respectively. Moreover, the 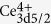 (885.69 eV), 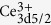 (887.93 eV), 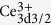 (907.35 eV) and 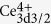 (917.2 eV) peaks have also been identified. As confirmed by the XRD results, the substitution of Ce ions result in the lattice expansion which leads to the escape of oxygen during high temperature sintering^29^. It can be inferred that the amount of Fe^2+^ ions and oxygen vacancies will increase with the substitution of Ce ions.

**Figure 3:**
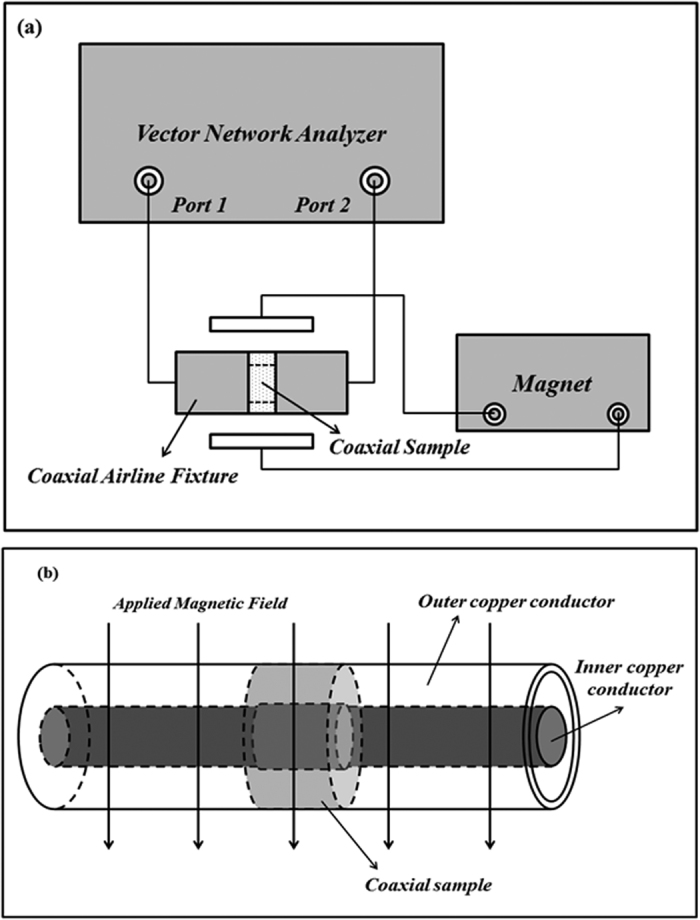
The schematic of MD effect measurement setup.

**Figure 4:**
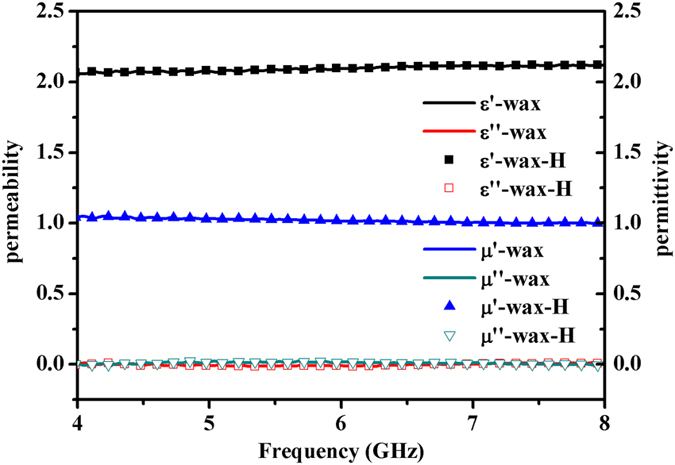
The electromagnetic properties of pure wax sample with (symbols) and without (lines) applied magnetic field H = ~1780 Oe.

In this report, the magnetodielectric response of *Ce*_*x*_*Y*_*3−x*_*Fe*_*5*_*O*_*12*_ *(x* = *0, 0.05, 0.1, 0.15, 0.2)* ferrites are detected by using an Agilent Technologies E5071C vector network analyzer (VNA) under the application of magnetic fields. The schematic of measurement setup is shown in Fig. 3. It is necessary to emphasize that the two magnetic poles of the magnet are aligned along the sides of the coaxial airline fixture so that the generated dc magnetic field can be made normal to the propagation direction of the microwave signal. By the transmission/reflection method, the accurate microwave electromagnetic properties of the samples can be obtained by an inversion calculation of the S parameters in VNA^30^. Since the dc magnetic field is superimposed upon the microwave signal, it is necessary to clarify whether the applied dc magnetic field will be affected by the microwave field. As a reference, Fig. 4 illustrates the electromagnetic properties of a pure wax coaxial sample with and without the applied dc magnetic field. The overlap of symbols and lines indicates that the permeability and permittivity of wax maintain about *1* and *2.1*, which is independent of the magnitude of the applied dc magnetic field. In other words, the measurement of electromagnetic properties will not be influenced by the application of the dc magnetic field.

**Figure 5:**
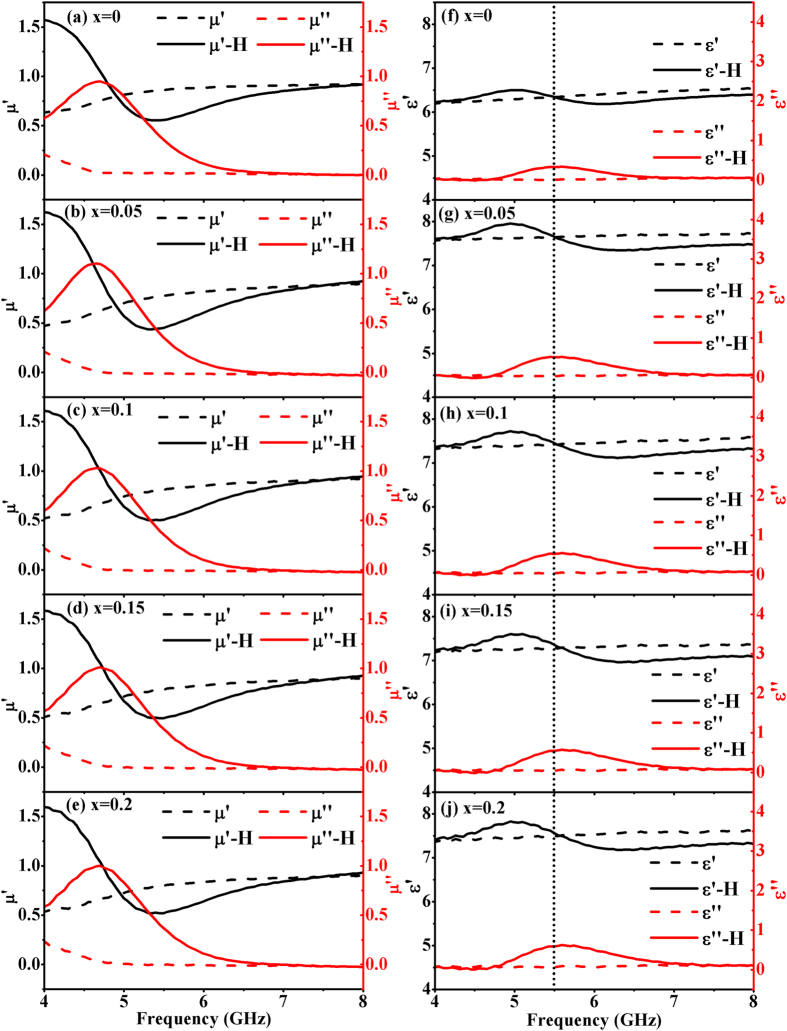
The electromagnetic properties of Ce-doped YIG ferrites without (dash lines) and with (solid lines) applied magnetic field H = ~1780 Oe.

Figure 5 shows the electromagnetic properties of *Ce*_*x*_*Y*_*3−x*_*Fe*_*5*_*O*_*12*_ *(x* = *0, 0.05, 0.1, 0.15, 0.2)* ferrites with and without the applied DC magnetic field. When the magnetic field is absent, the permeability of all the Ce-doped YIG samples (dash lines) remains largely unaltered with the substitution of Ce ions. In addition, the real part of the permeability gradually recovers to about 1 while the imaginary part decreases to near zero, which is a common phenomenon above the cut-off frequency caused by the internal magnetic anisotropy in the ferrites^31^. On the other hand, compared with the YIG/wax sample, all the Ce-doped YIG/wax samples reveal an obvious enhancement in permittivity from *6.1* to about *7.5* at C-band as shown in Fig. 5(f–j). It has been known that the dielectric relaxation polarization of YIG-based ferrites at microwave frequencies is associated with dipoles and interfacial polarization, whereas atomic and electronic polarizations can largely be ignored^32^,^33^. In Ce-doped YIG ferrites, it is assumed that the extra Fe^2+^ ions, arising from the substitution of Ce ions, may result in both hole-electron pairs and Fe^2+^-Fe^3+^ dipoles. It is worth noting that the substitution of Ce ions leads to an increase of Fe^2+^ ions while the total amount of Fe ions remains constant. As a result, more Fe^2+^-Fe^3+^ dipoles will be introduced by substitution of Ce ions. Therefore, the permittivity of Ce-YIG ferrites at C-band increases due to the enhancement of dipole polarization.

The MD behavior in *Ce*_*x*_*Y*_*3−x*_*Fe*_*5*_*O*_*12*_ *(x* = *0, 0.05, 0.1, 0.15, 0.2)* ferrites are presented in this work. Figure 5 also shows the permeability and permittivity spectra of *Ce*_*x*_*Y*_*3−x*_*Fe*_*5*_*O*_*12*_ *(x* = *0, 0.05, 0.1, 0.15, 0.2)* samples under the application of a dc magnetic field (solid lines). It is obvious that the resonance and relaxation phenomena appear to be visible in the permeability and permittivity spectra under the application of a dc magnetic field, respectively. The microwave magnetic field in the coaxial cable can naturally be divided into vertical h_⊥_ and horizontal h_∥_components, which is normal and parallel to the applied dc magnetic field, respectively. The ferromagnetic resonance (FMR) peak arises solely from the contribution of the vertical component h_⊥_, since the horizontal component h_∥_ is unable to respond to the alternating ac magnetization field^34^,^35^. On the other hand, an interesting observation is that the relaxation phenomena in permittivity emerge at frequencies corresponding to the FMR. That is, the location of dielectric relaxation peak is coherently related to the FMR over a narrow frequency band. For instance, the FMR frequency *f*_*0*_ of *Ce*_*x*_*Y*_*3−x*_*Fe*_*5*_*O*_*12*_ *(x* = *0.05)* sample is about 4.65 GHz that is near to its permittivity relaxation frequency *f*_*1*_ of 5.44 GHz. It can be inferred that the relaxation of the permittivity is a dynamic MD behavior that is strongly induced by FMR.

## Discussion

As illustrated in Fig. 5, both FMR phenomenon and the dynamic magnetodielectric relaxation process happen coherently and simultaneously in the Ce-doped YIG ferrite once a DC magnetic field is applied. It has been reported that MD coupling becomes more pronounced as the measurement is carried out in the vicinity of a resonance^36^. The interrelated dispersion phenomena emerging simultaneously in permeability and permittivity unveil a coupling of the magnetic and dielectric properties^37^. It is therefore inferred that the strong ferromagnetic resonance may give rise to a relaxation in the permittivity spectra, leading to an intrinsic magnetodielectric effect.

As mentioned, the FMR event gives rise to the relaxation in the permittivity spectrum due to the coupling of magnetic and dielectric properties. Additionally, the complex refractive index *n* of magnetic dielectric material can be described as:


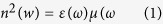


It can be found that the poles and zeros of *ε(ω)* and *μ(ω),* which may affect the dispersion type and resonance frequency, are reversed^38^. Additionally, it can be further inferred that the complex permittivity can be altered by permeability under certain conditions. As a result, a dielectric relaxation emerges as soon as strong FMR occurs in the permeability spectra due to the intrinsic coupling of magnetic and dielectric properties in the *Ce*_*x*_*Y*_*3−x*_*Fe*_*5*_*O*_*12*_ *(x* = *0, 0.05, 0.1, 0.15, 0.2)* ferrites. In essence, the MD effect originates from the intrinsic coupling between Fe^2+^ and Fe^3+^ ions, ultimately leading to enhanced polarization^39^. In ferrites, it is evident that dielectric relaxation primarily results from electronic hopping between Fe^2+^ and Fe^3+^ ions, and Fe^2+^-Fe^3+^ electric dipole^40^. As confirmed by the XPS spectra in Fig. 2, the coexistence of Fe^2+^ and Fe^3+^ ions in Ce-doped YIG ferrites may result in the formation of localized charged regions and dipoles. As soon as the dc magnetic field is applied, the Fe^2+^ and Fe^3+^ ions rearrange in response to the magnetic field by means of electron hopping between Fe^2+^ and Fe^3+^ ions^41^. Hence, the applied dc magnetic field leads to the reorientation of Fe^2+^-Fe^3+^ dipoles. It is believed that the electron fluctuation on the Fe site is the main contributor to the MD effect^39^. Alternatively, it has been known that the dipoles and interfacial polarization are the main contributors to the dielectric relaxation in ferrites at microwave frequencies, whereas atomic and electronic polarization can be largely ignored^32^,^33^. As known, the FMR phenomenon is an energy absorption process, whereas a dynamic magneto-dielectric interaction also reflects conversion or consumption of electromagnetic wave energy. Therefore, the dynamic MD behavior induced relaxation dispersion phenomenon occurs in the permittivity spectrum since the rearrangement and reorientation of Fe^2+^-Fe^3+^ dipoles in Ce-doped YIG ferrite lags behind the alternating electric field of high frequency electromagnetic wave. In other words, a dynamic MD effect originated from the rearrangement or reorientation of Fe^2+^–Fe^3+^ dipoles gives rise to the consumption of electromagnetic wave energy at microwave frequencies, which is observed in terms of a relaxation phenomenon in the permittivity spectrum.

Both the FMR phenomenon and magnetodielectric effect are associated with energy absorption. However, it is inferred that visible energy absorption due to the FMR or MD effect might be measurable at different frequencies, which is determined by the distinct loss mechanisms. In present work, the energy absorption of the Ce-doped YIG ferrite can be calculated by the S parameters measured by the VNA as the following equation:


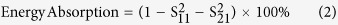


**Figure 6:**
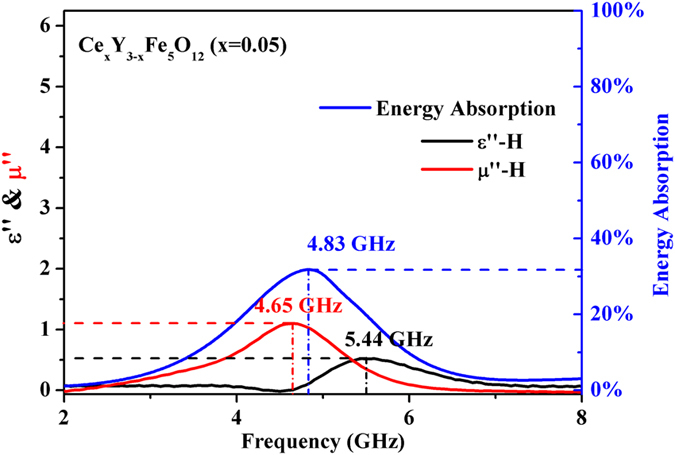
The dependence of imaginary part of permeability and permittivity & energy absorption on the frequency in *Ce*_*x*_*Y*_*3−x*_*Fe*_*5*_*O*_*12*_ *(x* = *0.05)* ferrite with H = 1780 Oe.

Figure 6 depicts the energy absorption of *Ce*_*x*_*Y*_*3−x*_*Fe*_*5*_*O*_*12*_ *(x* = *0.05)* ferrite when a magnetic field of *1780 Oe* is applied. It can be found that the peak of total energy absorption is located at 4.83 GHz, whereas FMR frequency of Ce-doped YIG *(x* = *0.05)* sample is located at 4.65 GHz. As it has been known, the FMR is a forceful energy absorption phenomenon in the microwave frequency band and the energy will finally be consumed by lattice vibrations through the coupling of phonons. In Ce-doped YIG ferrites, it can be inferred that part of the energy absorbed through FMR is the energy source of the rearrangement and reorientation of Fe^2+^-Fe^3+^ dipoles caused by the applied dc magnetic field^42^. Due to the intense energy absorption inherent in the FMR, this resonance can be considered as the most effective way for extra energy to be communicated to the system and enhance the intrinsic magnetic and dielectric losses of the Ce-doped YIG ferrites. It is predictable that the width of the total energy absorption peak is tailored by both FMR and MD effects, which is in complete agreement with experimental data. Furthermore, compared with the FMR, the dielectric dispersion caused by the dynamic MD effect is relatively weaker since the MD effect is a weak coupling relationship between permeability and permittivity in single-phase materials. Hence, the peak of energy absorption locates at the frequency that is much closer to the FMR frequency since the dielectric relaxation caused by dynamic MD behavior is a secondary factor of energy absorption.

**Figure 7:**
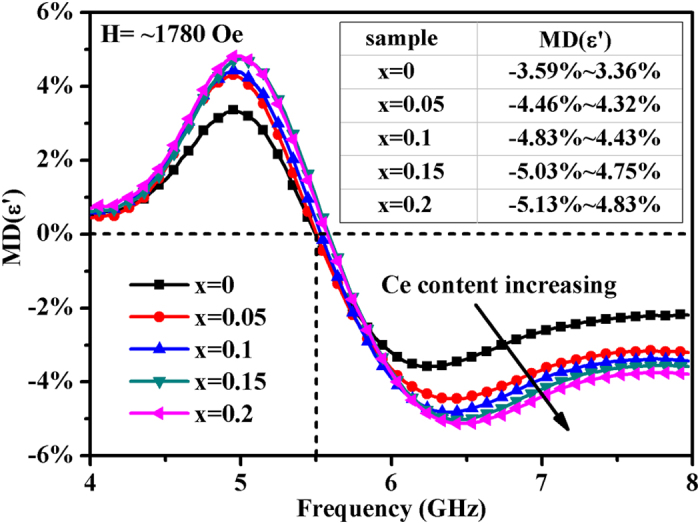
The magnetodielectric responses of Ce-doped YIG ferrites.

The MD coefficients of *Ce*_*x*_*Y*_*3−x*_*Fe*_*5*_*O*_*12*_ *(x* = *0, 0.05, 0.1, 0.15, 0.2)/wax* samples are shown in Fig. 7. The MD responses of all samples display relaxation characteristics as their permittivity spectra do. It is noteworthy that all the MD responses of samples become near zero in the vicinity of *f* = *5.5* *GHz*, but exhibit a maximum effect at a frequency slightly lower or higher than *5.5* *GHz*. Moreover, it can be observed that the relaxation also occurs near *5.5* *GHz,* which indicates the MD coefficients reveal relative variations in permittivity due to dielectric relaxation induced by FMR. The amplitude of the MD responses at C-band increases with Ce ions, as depicted in the insert table of Fig. 7. It can be inferred that the Fe^2+^ ions will increase with the substitution of Ce ions while the total amount of Fe ions remains unchanged. Therefore, we conjecture that the increase of Ce ions may introduce more Fe^2+^-Fe^3+^ dipoles in the ferrite, which enhances the electron exchange between Fe^2+^ and Fe^3+^ ions. An enhanced electron exchange may intensify the polarizability due to more intense electron fluctuations and consequently enhances the MD effect. In the current work, the strongest MD response is measured to be −*5.13%*~+*4.83%* for *x* = *0.2* sample in the relaxation type dispersion region, which is considerably larger than the MD effect previously reported at microwave frequencies.

In conclusion, we have fabricated *Ce*_*x*_*Y*_*3−x*_*Fe*_*5*_*O*_*12*_ *(x* = *0, 0.05, 0.1, 0.15, 0.2)* ferrites by traditional solid-state reaction method. The emergence of Fe^2+^ ions due to the substitution of Ce ions affects the electromagnetic properties of the Ce-doped YIG ferrites. We propose that the electron fluctuations on Fe sites are responsible for the ferromagnetic resonance induced large room temperature C-band magnetodielectric effect in the Ce-doped YIG ferrites. In addition, the intensity of the MD response increases with the substitution content of Ce ions. We believed that the investigation of room temperature MD coupling in single-phase microwave materials will prove enabling to the development of next generation multifunctional adaptive materials, devices, and integrated microwave circuits.

## Methods

In the experiments reported here, polycrystalline ferrite samples of *Ce*_*x*_*Y*_*3−x*_*Fe*_*5*_*O*_*12*_ *(x* = *0, 0.05, 0.1, 0.15, 0.2)* were prepared by traditional solid-state reaction method. Powders of *Y*_*2*_*O*_*3*_ *(99.9%), Fe*_*2*_*O*_*3*_ *(99.9%), CeO*_*2*_ *(99.9%)* were mixed in appropriate stoichiometric ratios through ball-milling for 4 hours and the dried mixtures were calcined at 1473 K for 4 hours. Some calcined powders were then ground by ball-milling a second time for 4 hours, and then pressed into green pellets with 6 wt. % of polyvinyl alcohol (PVA) binder. The rest of calcined powders and the green pellets were then sintered at 1723 K for 4 hours in air. The sintered powders were finally pressed with 10 wt% wax into coaxial toroidal samples (inner diameter *d* = *3* *mm*, outer diameter *D* = *7* *mm* and height *h* = *3* *mm*) for the measurement of their microwave electromagnetic properties.

The crystalline phases of sintered samples were identified by powder X-ray diffraction (XRD). The elemental spectra of *Ce*_*x*_*Y*_*3−x*_*Fe*_*5*_*O*_*12*_ *(x* = *0, 0.05, 0.1, 0.15, 0.2)* were measured by X-ray photoelectron spectroscopy (XPS). The microwave electromagnetic properties and magnetodielectric phenomena of Ce-doped YIG ferrites were detected using an Agilent Technologies E5071C vector network analyzer (VNA) with a DC magnetic field applied in the frequency range of *4*–*8* *GHz*. The magnetodielectric coefficient can be described by the following formula:


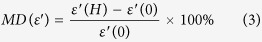


where ε’(H) and ε’(0) represent the real part of the complex permittivity with and without applied DC magnetic field, respectively.

## Additional Information

**How to cite this article**: Chen, F. *et al.* Ferromagnetic resonance induced large microwave magnetodielectric effect in cerium doped Y_3_Fe_5_O_12_ ferrites. *Sci. Rep.* **6**, 28206; doi: 10.1038/srep28206 (2016).
